# Supplementary material for: Social Avoidance of Mice in Pain in Naturalistic Conditions
Source: Affect Sci. 2024 Sep 23;6(1):159–70. doi: 10.1007/s42761-024-00276-8 (PMC11903981; doi:10.1007/s42761-024-00276-8)
Supplement: Supplementary file 1 — (DOCX 1.14 MB) [file 42761_2024_276_MOESM1_ESM.docx]

**
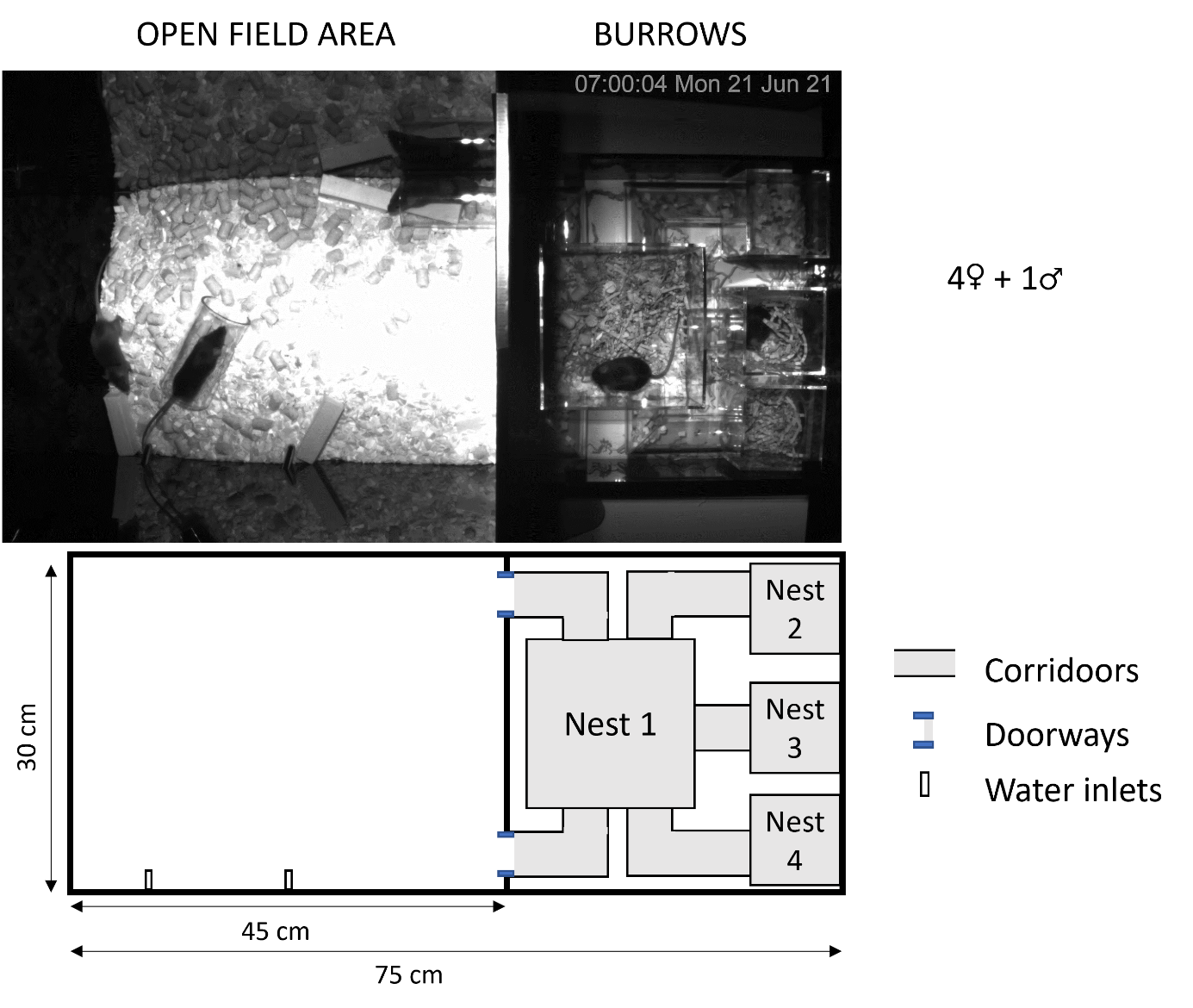
**

**Figure S1**. Picture of the seminatural environment (SNE) (top). Scheme of the SNE (bottom). Five mice (4 females and 1 male) are placed in the SNE for 7 days (day 0 to 6) with food and water available *ad libitum*.

**
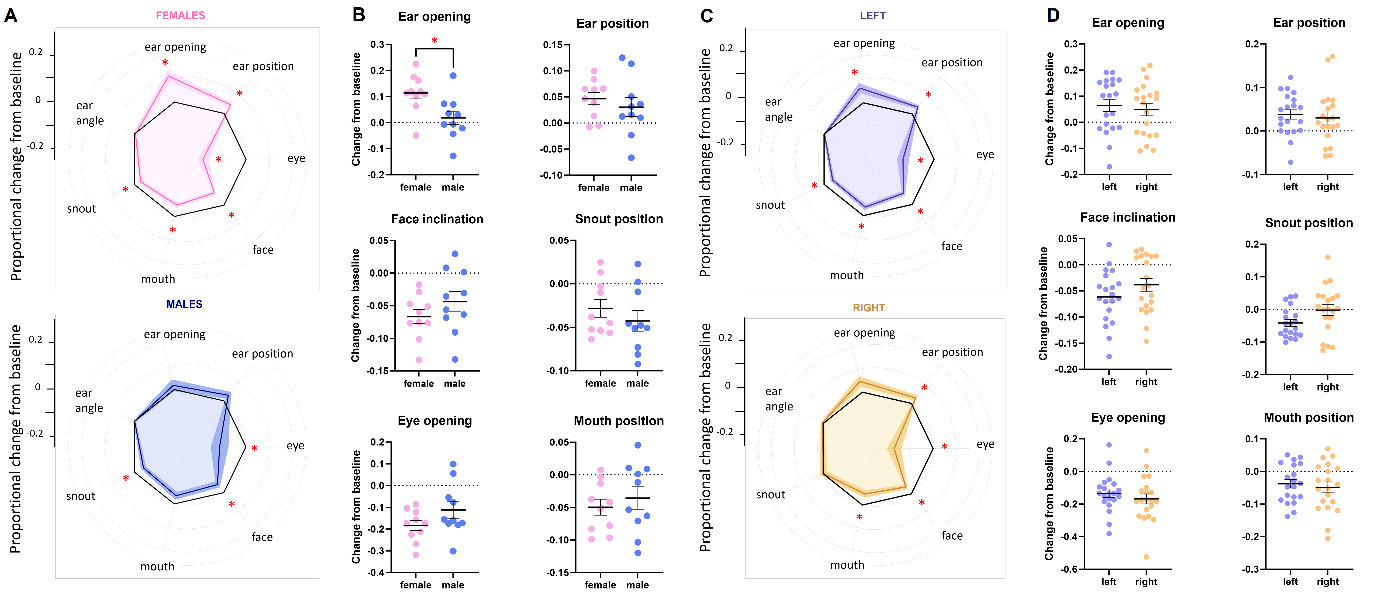
**

**Figure S2.** Facial expressions elicited by formalin injection (neck and hind paw data pooled, n = 10 females + 10 males). A. Female (pink) and male (blue) response profile to formalin (30 min observation). B. Sex difference in facial parameters’ change from baseline. C. Responses profiles to formalin produced by pictures from the left (purple) and right (yellow) side of mouse faces. D. Side difference in facial parameters’ change from baseline. Data are mean ± SEM and individual data points. Response profiles (A-C): one-sample *t*-test; Scatter plots (B-D): *t*-test. *, p < 0.05.


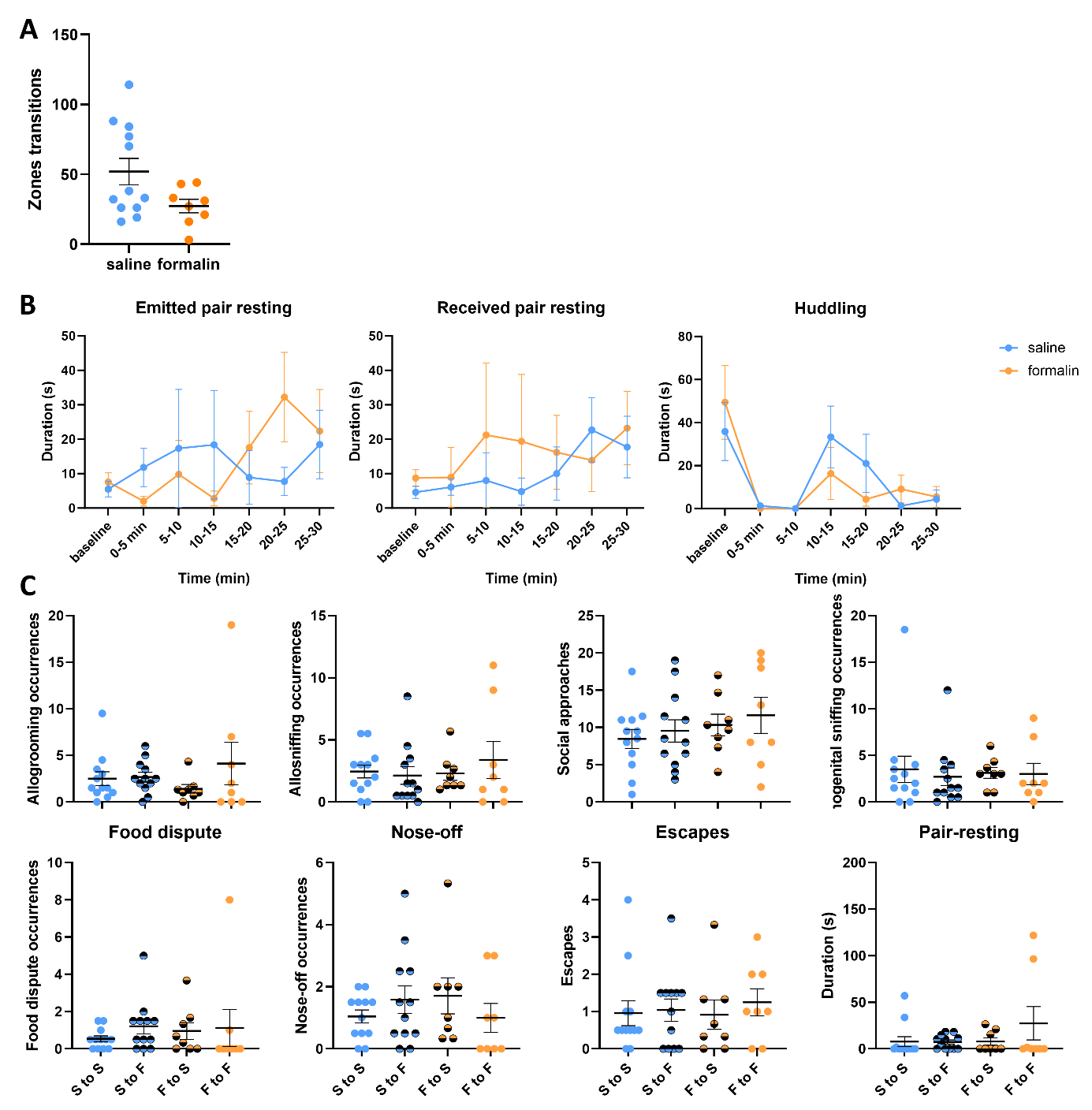


**Figure S3**. **A**. Zone transitions in the SNE, *t*-test **B**. Emitted and received resting behaviors. Two-way ANOVAs for repeated measured on the time interval factor, *, p < 0.05. Data are mean ± SEM. **C**. Dyadic interactions at baseline from saline to saline (S-S, blue, n=12), saline to formalin (S to F, half blue, n=12), formalin to saline (F to S, half yellow, n=8) and formalin to formalin (F to F, yellow, n=8) mice. One-Way ANOVAs and Kruskal-Wallis rank tests, all ps > 0.289.

**
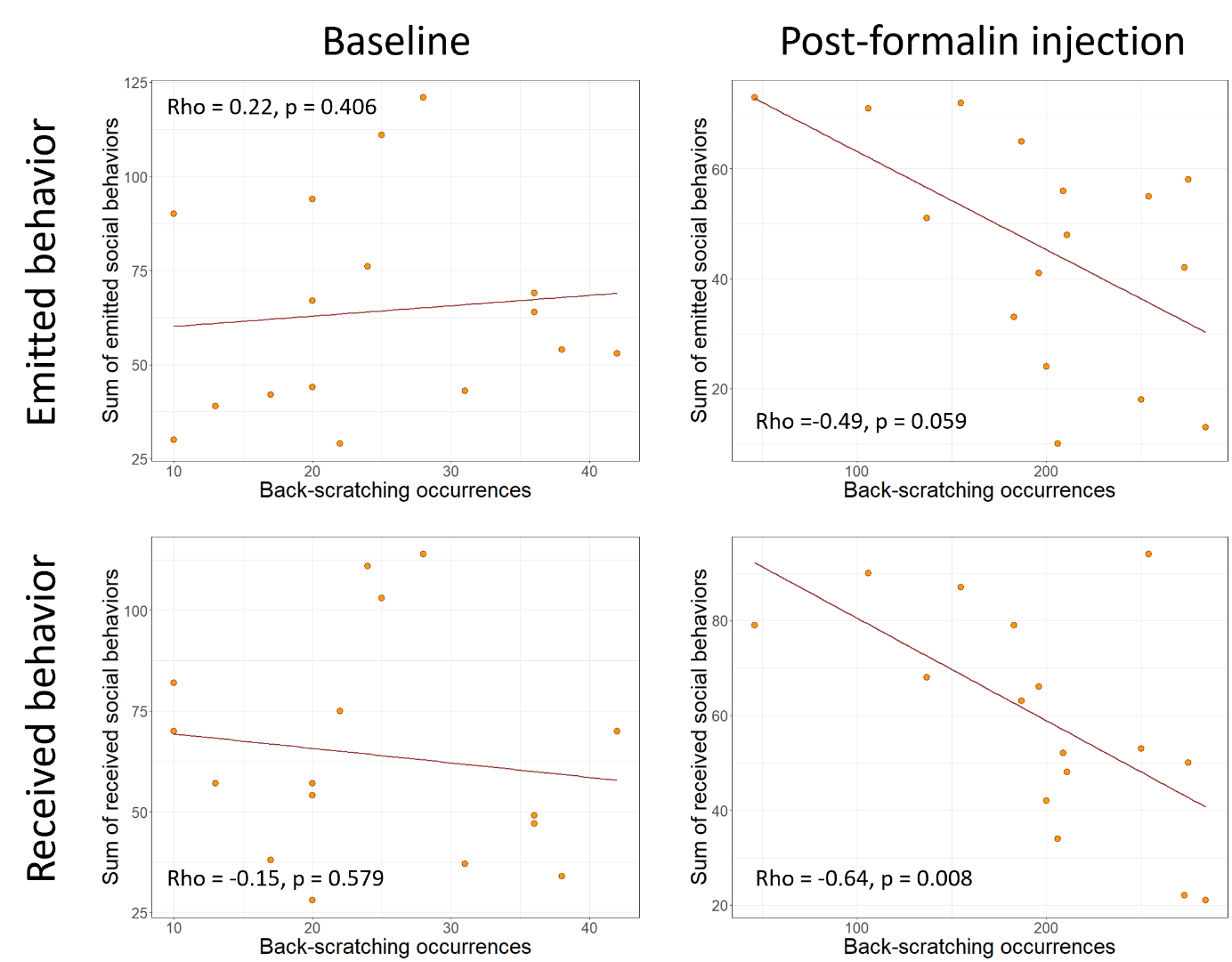
**

**Figure S4**. Spearman’s correlations between occurrences of back-scratching and the sum of emitted (top row) or received (bottom row) social behavior occurrences at baseline (left) or following formalin injection (right). Included social behaviors are allogrooming, allosniffing, anogenital sniffing, social approach and pair-resting. N=16 formalin-treated mice (8 during the diurnal and 8 during the nocturnal phase of the light cycle).
